# Evolutionary and Molecular Analysis of Complete Genome Sequences of Norovirus From Brazil: Emerging Recombinant Strain GII.P16/GII.4

**DOI:** 10.3389/fmicb.2020.01870

**Published:** 2020-08-06

**Authors:** Juliana Merces Hernandez, Luciana Damascena Silva, Edivaldo Costa Sousa Junior, Jedson Ferreira Cardoso, Tammy Kathlyn Amaral Reymão, Ana Caroline Rodrigues Portela, Clayton Pereira Silva de Lima, Dielle Monteiro Teixeira, Maria Silvia Souza Lucena, Marcio Roberto Teixeira Nunes, Yvone Benchimol Gabbay

**Affiliations:** ^1^Postgraduate Program in Biology of Infectious and Parasitic Agents, Institute of Biological Sciences, Federal University of Pará, Belém, Brazil; ^2^Virology Section, Evandro Chagas Institute, Brazilian Ministry of Health, Ananindeua, Brazil; ^3^Center for Technological Innovation, Evandro Chagas Institute, Brazilian Ministry of Health, Ananindeua, Brazil

**Keywords:** norovirus, GII.P16/GII.4, emergent recombinant, gastroenteritis, molecular evolution, complete genome

## Abstract

Noroviruses (NoVs) are enteric viruses that cause acute gastroenteritis, and the pandemic GII.4 genotype is spreading and evolving rapidly. The recombinant GII.P16/GII.4_Sydney strain emerged in 2016, replacing GII.P31/GII.4_Sydney (GII.P31 formerly known as GII.Pe) in some countries. We analyzed the complete genome of 20 NoV strains (17 GII.P31/GII.4_ Sydney and 3 GII.P16/GII.4_Sydney) from Belém and Manaus, Brazil, collected from 2012 to 2016. Phylogenetic trees were constructed by maximum likelihood method from 191 full NoV-VP1 sequences, demonstrated segregation of the Sydney lineage in two larger clades, suggesting that GII.4 strains associated with GII.P16 already have modifications compared with GII.P31/GII.4. Additionally, the Bayesian Markov Chain Monte Carlo method was used to reconstruct a time-scaled phylogenetic tree formed by GII.P16 ORF1 sequences (*n* = 117) and three complete GII.P16 sequences from Belém. The phylogenetic tree indicated the presence of six clades classified into different capsid genotypes and locations. Evolutionary rates of the ORF1 gene of GII.P16 strains was estimated at 2.01 × 10^–3^ substitutions/site/year, and the most recent common ancestors were estimated in 2011 (2011–2012, 95% HPD). Comparing the amino acid (AA) sequence coding for ORF1 with the prototype strain GII.P16/GII.4, 36 AA changes were observed, mainly in the non-structural proteins p48, p22, and RdRp. GII.P16/GII.4 strains of this study presented changes in amino acids 310, 333, 373, and 393 of the antigenic sites in the P2 subdomain, and ML tree indicating the division within the Sydney lineage according to the GII.P16 and GII.P31 polymerases. Notably, as noroviruses have high recombination rates and the GII.4 genotype was prevalent for a long time in several locations, additional and continuous evolutionary analyses of this new genotype should be needed in the future.

## Introduction

Noroviruses (NoVs) are enteric viruses belonging to the *Caliciviridae* family that commonly cause pandemic acute gastroenteritis worldwide (Pires et al., 2015). The global burden of illness is estimated to be 570.3 million cases in low- and middle-income countries (Bartsch et al., 2016). In countries such as Brazil, the risk factors for noroviruses can be five times more than those for other enteropathogens (Lima et al., 2019). NoV saffect all ages, but severe disease is particularly observed in children (less than 5 years old), elderly people, and immunocompromised individuals (Cardemil et al., 2017; Woodward et al., 2017; Riera-Montes et al., 2018).

NoVs are genetically diverse and classified into at least 10 genogroups (GI-GX) and more than 45 genotypes (ICTV Virus Taxonomy, 2020). Despite the wide variability in NoVs, the GII genogroup is responsible for most cases in humans, especially the GII.4 genotype, which has been most associated with outbreaks and sporadic cases of viral gastroenteritis. The viral genome is a single-stranded positive-sense RNA genome of approximately 7.6 kb with a polyA tail. It is organized in three open reading frames (ORF1-3) (Green, 2013). ORF1 encodes a large polyprotein that after cleavage forms six non-structural proteins, including the viral RNA-dependent RNA polymerase (RdRp) involved in replication. ORF2 encodes the major capsid protein (VP1) structurally comprised of two domains, shell (S) and protruding (P). The P domain is subdivided into the P1 and P2 subdomains. Antigenic sites and carbohydrates binding-sites map into the P2 subdomain, both playing a major role in susceptibility to norovirus infection (Lindesmith et al., 2008). ORF3 encodes a minor capsid protein (VP2).

Norovirus presents multiple genotypes, but GII.4 strains are the predominant worldwide. GII.4 noroviruses have been shown the emergence of variants that present changes in antigenic sites, which facilitate escape from herd immunity elicited by previous variants (Debbink et al., 2012; Tohma et al., 2019).

In addition, Lindesmith et al. (2018), reported a strategy of camouflaging antigenic sites to carbohydrate blocking antibodies, named “particle breathing.” Since 1995, many variants causing global epidemics have emerged, including US 1995/96 in 1996 (Fankhauser et al., 1998), Farmington Hills in 2002 (Lopman et al., 2004), Hunter in 2004 (Bull et al., 2006), Den Haag in 2006 (Eden et al., 2010), New Orleans in 2009 (Yen et al., 2011), and Sydney in 2012 (van Beek et al., 2013). However, in 2014, a non-GII.4 genotype, named GII.17_Kawasaki, was reported as the main cause of outbreaks in Asia (Lu et al., 2015; Matsushima et al., 2015; Thanh et al., 2016), some countries of the Americas (Parra and Green, 2015; Degiuseppe et al., 2017; Silva et al., 2017) and Europe (De Graaf et al., 2015; Giammanco et al., 2017).

The GII.4_Sydney lineage emerged in 2012, and still causes a large number of cases. In 2016, the variant GII.4_Sydney recombined in association with a novel GII.P16 ORF1, and the circulation of this variant increased in many countries such as Germany, South Korea and United States (Choi et al., 2017; Niendorf et al., 2017; Barclay et al., 2019). In Brazil, this novel variant was identified in 59.5% (22/37) of genetically characterized samples (Barreira et al., 2017). Additionally, the increase in norovirus activity at the end of 2016 to 2017 may have occurred because of the emergent GII.P16/GII.2 recombinant (Fu et al., 2017; Kwok et al., 2017; Liu et al., 2017). This GII.P16 genotype presented several amino acid changes in non-structural proteins, such as RdRp, and these changed seemed to facilitate its transmission (Cannon et al., 2017; Tohma et al., 2017; Cheung et al., 2019). In this study, we analyzed the complete genomes of GII.4 strains collected over 5 years to understand their genetic characteristics and investigate the emergence of GII.P16/GII.4.

## Materials and Methods

### Clinical Samples

This study on the emergence of the GII.P16/GII.4 strain started from previous data from studies conducted with samples from the cities Belém and Manaus in northern Brazil (Hernandez et al., 2018; Reymão et al., 2018).

Stool samples analyzed in this study were collected between 2012 and 2016. From January 2010 to December 2016, 32.8% (557/1698) of the samples from the study region were positive for norovirus by the EIA test. GII.4 was the genotype most frequently detected, with the emergence of the GII.P31/GII.4-Sydney 2012, GII.17_2014 and GII.P16/GII.4 strains. Reports about GII.P31/GII.4 and GII.17_2014, as well as the temporal distribution and emergence period of these strains, have already been described (Silva et al., 2013; Hernandez et al., 2018; Reymão et al., 2018; Costa et al., 2019).

Considering the information available on norovirus epidemiological surveillance, 28 samples were selected for whole genome sequencing. The samples were previously characterized as the genotype GII.P31/GII.4 as well as three samples as GII.P16/GII.4 by direct sequencing of short genomic fragments (from 200 to 250 bp). To provide temporal representation, the samples were distributed by year and State, as shown in Table 1.

**TABLE 1:** Data about the samples selected for this study.

**Year of collection**	**Local (City/State)**	**Norovirus genotype**
2012	*N* = 3 (Belém/Pará)	GII.P31/GII.4
	*N* = 1 (Manaus/Amazonas)	GII.P31/GII.4
2013	*N* = 3 (Belém/Pará)	GII.P31/GII.4
	*N* = 3 (Manaus/Amazonas)	GII.P31/GII.4
2014	*N* = 3 (Belém/Pará)	GII.P31/GII.4
	*N* = 3 (Manaus/Amazonas)	GII.P31/GII.4
2015	*N* = 3 (Belém/Pará)	GII.P31/GII.4
	*N* = 2 (Manaus/Amazonas)	GII.P31/GII.4
2016	*N* = 6 (Belém/Pará)	GII.P31/GII.4 (*N* = 3); GIIP.16/GII.4 (*N* = 3)
	*N* = 1 (Manaus/Amazonas)	GII.P31/GII.4
Total	28 (Belém = 18, Manaus = 10)	GII.P31/GII.4 (*N* = 25); GIIP.16/GII.4 (*N* = 3)

### Ethics Approval

This study was approved by the Ethics Committee on Human Research of Evandro Chagas Institute, Brazilian Ministry of Health (protocol No. 0039/2011 and protocol No. 0017/2014 update No. 1.318.103 of 2015) as described by Hernandez et al. (2018) and Reymão et al. (2018).

### Viral Genome Extraction and Sequencing

A fecal suspension (10% w/v) prepared in Tris/HCl/Ca^2+^ buffer was used for nucleic acid extraction by a QIAamp viral RNA mini kit (Qiagen, Hilden, Germany) according to the manufacturer’s guidelines or using the silica method (Hernandez et al., 2018). The extracted RNA was quantified by a Qubit 2.0 fluorometer using the Qubit RNA BR assay kit (Thermo Fisher). Then, the samples were subjected to reverse transcription and purified using a cDNA synthesis system kit (Roche, Branford, CT, United States) to obtain pure double-stranded DNA. The DNA was quantified using the Qubit DNA BR assay kit (Thermo Fisher Scientific) and analyzed for fragmentation and quality profile using an Agilent 2100 Bioanalyzer (Agilent Technologies, Santa Clara, CA, United States) as recommended by the manufacturer. Subsequently, libraries for sequencing were prepared using the Illumina Nextera XT DNA Library Prep kit and sequenced on an Illumina HiSeq 2500 instrument (Illumina, San Diego, CA, United States) with the high-output V4 2 × 100-bp sequencing kit.

### Identification, Assembly, and Genome Annotation

The adapters were removed from raw sequence reads using Trim Galore pipeline v.0.4.5^1^, and the reads were quality-trimmed and filtered by Prinseq-lite (Schmieder and Edwards, 2011). Reads were assembled *de novo* by IDBA-UD assembler v.1.1.3 (Peng et al., 2012). Additionally, the 3′ and 5 ends were verified using reference mapping based on norovirus prototype strains obtained from GenBank. Genome management and annotation were conducted in Geneious 9.1.2 (Kearse et al., 2012). The whole sequences of norovirus were deposited in GenBank under the accession numbers MN525275, MN525276 and MT474045/MT474046 for the GII.P16/GII.4 strains and MT474032-MT474044/MT474047- MT474052 for the GII.P31/GII.4 strains.

### Phylogenetic Analyses of Capsid

The complete norovirus ORF2 sequences from this study were aligned with a database composed by 1369 genomes obtained from all norovirus GII.4 sequences of GenBank. To remove redundant sequences, CD-HIT was applied, resulting in 947 sequences. After removing identical sequences using as cut-off based on the e-value of the classification in groups with CD-HIT, 191 genomes had full VP1 sequences that were used to reconstruct the phylogeny. All sequences were aligned with Mafft (Katoh and Standley, 2013), and maximum likelihood phylogenetic analyses based on GTR nucleotide substitution model were performed using RAxML v.8.2 with 1,000 bootstraps (Stamatakis, 2014). Tree editing was performed with Evolview (Zhang et al., 2012). To confirm the clades within GII.4_Sydney, we performed another ML tree, including all GII.4 variants with GII.P31, GII.P16 and GII.P4 polymerases, totalizing 342 sequences of diverse sizes. The parameters were the same previously described for RaxML.

### Evolutionary History of Recombinant NoV GII.P16

To construct a time-scaled phylogenetic tree, a dataset formed of full or near-full GII.P16 ORF1 sequences (*n* = 117) available in GenBank combined with three complete GII.P16 sequences from Belém was generated. The dataset was subjected to Bayesian Markov Chain Monte Carlo (MCMC) analysis implemented in BEAST software v1.10.8 (Drummond et al., 2012). GTR + I + G was the nucleotide substitution model used. To choose the most appropriate molecular clock and the spatial model, several models were tested, and higher values of path sampling (ps) and stepping stone (ss) indicated the best model fit. A final clock ran in triplicate with 50 million generations.

### P2 Region Analysis

The P2 subdomain of four GII.P31/GII.4 and two GII.P16/GII.4 strains were analyzed to explore amino acid changes in antigenic sites of the VP1 protein using the Geneious 9.1.2 (Kearse et al., 2012).

## Results

Twenty complete or near complete genomes of norovirus strains were obtained from the samples sequenced using Illumina HiSeq. An average of 11,510,081 reads were generated per sample. The number of reads and the average coverage of each base for NoV is shown in Supplementary Table S1. The sequences generated presented 7,503 nt in length on average [excluding the poly(A) tail]. The open reading frames (ORFs) were 5,094 (ORF1), 1,623 (ORF2), and 807 nt (ORF3) in length.

**FIGURE 1:**
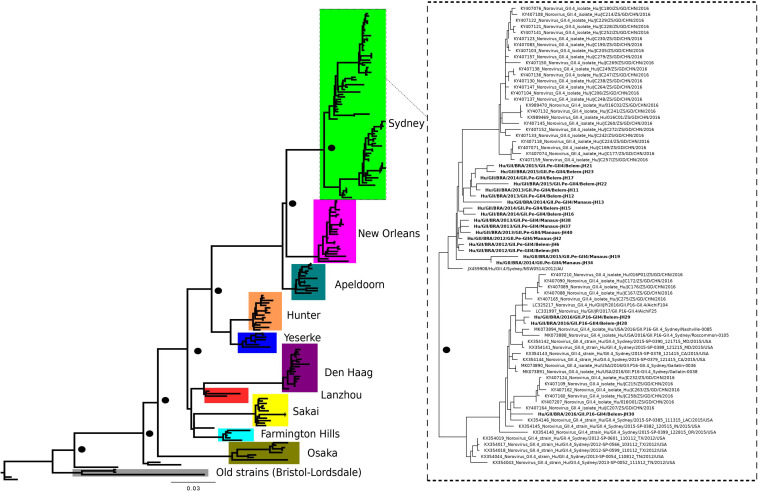
Phylogenetic tree constructed by maximum likelihood method based on the complete ORF2 gene (1,623 bp) of 191 nucleotide sequences norovirus GII.4 variants. The color of the branches is based on the NoV variants. Bootstrap values equal to or more than 90% are represented in black circles.

**FIGURE 2:**
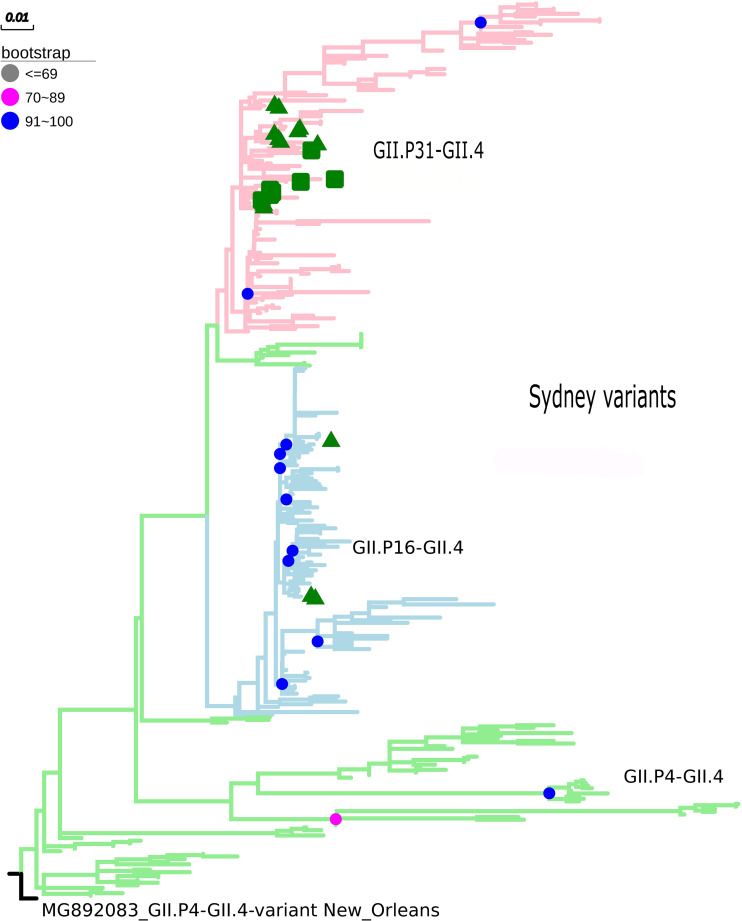
Phylogenetic tree constructed by maximum likelihood method based on the ORF2 gene of 342 nucleotide sequences of norovirus GII.4 (GII.P31, GII.P16, and GII.4 polymerases). Bootstrap values equal to or more than 90% are represented in blue circles. The pink clades represent the GII.P31/GII.4 variant (GII.P31 formerly known as GII.Pe), the blue clades represent the GII.P16/GII.4 strains, and the green clades represent the GII.P4/GII.4 strains. The green triangle indicates samples from the city of Belém, Pará and the green square for samples from the city of Manaus, Amazonas. The outgroup used was the New Orleans strain (MG892083).

The maximum likelihood phylogenetic tree of the complete ORF2 gene (1,623 nt) demonstrated that the epidemic strain segregated in two larger clades within the Sydney lineage, collected from 2012 to 2016. The GII.P16/GII.4 sequences of this study clustered with other emerging recombinants from China and the United States (Figure 1). Sequence analysis in BLASTn demonstrated that these sequences had 98.8 and 98.6% nucleotide identity with other GII.P16/GII.4 recombinant strains from the United States (MK764013) and United Kingdom (KY887601), respectively. High nucleotide identity (>98%) was also observed with the first report of a complete genome sequence of norovirus GII.P16/GII.4, isolated in Japan (Matsushima et al., 2016). The ML tree with large number of sequences confirms the division within the Sydney lineage, according to the GII.P16 and GII.Pe polymerases (Figure 2). The GII.4_Sydney strains that have the GII.P16 polymerase form an independent clade of the other Sydney variants.

**FIGURE 3:**
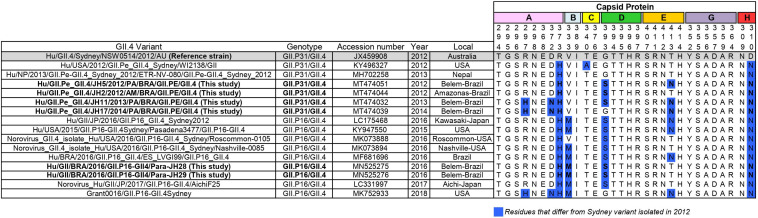
Alignment of antigenic residues of major capsid protein (P2 region) of GII.P31/GII.4 (GII.P31 formerly known as GII.Pe) and GII.P16/GII.4 strains. Residues mapping on previously characterized A-H epitopes. The prototype strain representative of GII.P31/GII.4 variant that emerged 2012 is highlighted in gray. Amino acid residues that differ from those of the prototype is highlighted in blue. Sequences of this study are in bold.

The capsid gene of this new recombinant (GII.P16/GII.4) shared 96.8% nucleotide identity with the capsid gene of the GII.4_Sydney prototype isolated in 2012 (JX459908) and 96.4% with GII.4[P31] from the same city of Brazil (MT474032), indicating that the nucleotide substitutions accumulated over the years, as expected for norovirus strains. GII.P16/GII.4 strains of this study presented changes in amino acids 310, 333, 373, and 393 and of the antigenic sites in the P2 subdomain (Figure 3).

The GII.P31/GII.4_Sydney sequence isolates in this study showed a high degree of pairwise identity (more than 98%) with Japanese (AB972473), Brazilian (MN307994, MN308017), and American (KY486271) strains. BLAST analysis showed that the capsid gene sequence (1,623 bp) shared 97.2–99% identity with the Sydney prototype isolate obtained in 2012 (JX459908).

**FIGURE 4:**
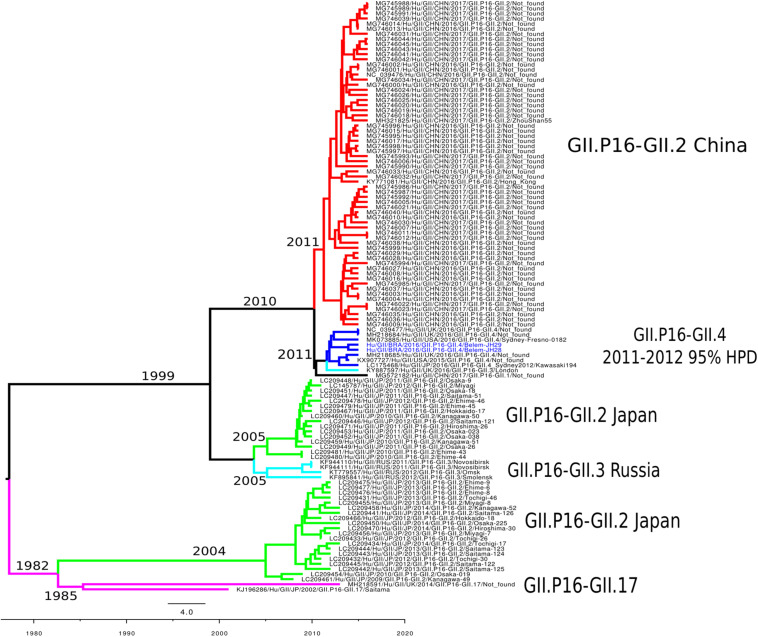
Time-scaled phylogenetic tree of the 117 sequences based on the whole nucleotide sequence of the ORF1 gene of human norovirus GII.P16 genotype strains. The tree was constructed using the Bayesian Markov Chain Monte Carlo method (MCMC) and the GTR + I + G nucleotide substitution model. Branches are colored according to the distinct norovirus genotype. Norovirus strains isolated in this study are colored blue. The scale bar represents the unit of time (years).

To reconstruct the time-scaled phylogenetic tree of the ORF1 gene (5,100 nt) for the RdRp of norovirus GII.P16, the Bayesian MCMC method was used with the best model lognormal GMRF Bayesian Skyrideon a strict clock. The path sampling (PS) and stepping-stone sampling (SS) parameters used in the evolutionary analysis are shown in Supplementary Table S3. The phylogenetic tree formed six clades classified into different capsid genotypes and locations. The cluster GII.P16/GII.4 was closer to the GII.P16/GII.2 Chinese strains. Notably, GII.P16-GII.2 re-emerged in Japan (2013–2014) with nucleotide modifications. Furthermore, the cluster GII.P16-GII.4_Sydney_2012 contained samples from the United Kingdom, United States and Japan described in 2016, including the LC175467 prototype (Matsushima et al., 2016; Figure 4). The evolutionary rates of the ORF1 gene of norovirus GII.P16 strains was estimated at 2.01 × 10^–3^ substitutions/site/year. Moreover, the most recent common ancestor (TMRCA) of GII.P16 strains was estimated in 2011 (2011–2012, 95% HPD) (Figure 4).

In addition, comparison of the amino acid (AA) sequence coding for ORF1 with the sequence of the prototype strain GII.P16/GII.4 (LC175468) identified 36 AA changes in the ORF1 gene, 11 of which had not yet been described in the literature (Supplementary Table S2). We found mutations in non-structural proteins such as p48, p22, and RdRp. Additionally, eleven of these non-synonymous changes modified the chemical nature and polarity of the AAs (Supplementary Table S2).

## Discussion

Here, we demonstrated the evolutionary and molecular analysis of GII.4 strains detected in the states of Pará and Manaus, Brazil. In Brazil, the epidemiology of the norovirus GII.4 strain has been well established in recent years. Previous studies conducted by Hernandez et al. (2018) and Reymão et al. (2018) demonstrated the emergence of the GII.P31/GII.4_Sydney lineage in 2012 and its high frequency from 2012 to 2016. Also, Fioretti et al. (2014) observed the rapid evolutionary dynamics in GII.4 variants in several regions of Brazil.

GII.4_Sydney variant predominated for several years with the GII.P31 (GII.Pe) polymerase. However, in 2015–2016 emerged as GII.P16/GII.4_Sydney recombinant. In the same period, it was observed the co-circulation of all circulating pandemic strains related worldwide in samples from Northern region of Brazil, including the GII.P16/GII.4 (Costa et al., 2019). In addition, Barreira et al. (2017) detected GII.P16/GII.4 circulating in the Southeastern Brazilian coast state among children up 11 years old. Therefore, this study was conducted to investigate the molecular features of NoV-GII.4 strains in two states of the Northern region of Brazil, to better understand the circulation of these pandemic viruses in this country.

The GII.P16/GII.4 strain emerged in the Amazon region in 2015 and was detected in 16.9% (16/95) of the cases during 2015–2016, being the second most prevalent virus since GII.P31/GII.4, which corresponded to 50.5% (48/95) of the NoV-positive cases (Costa et al., 2019). In this study, nucleotide analysis by BLASTn indicated that the strains circulating in Brazil had high identity with sequences of GII.P16/GII.4 from the United States (Barclay et al., 2019) and United Kingdom (Ruis et al., 2017). These data suggest a possible importation of this strain from other countries, considering previous descriptions about the circulation of this genotype in other places.

Several studies have investigated the evolutionary pattern of norovirus, especially using partial regions of the genome and the VP1 gene (Qiao et al., 2016; Parra et al., 2017; Tohma et al., 2018, 2019). However, a very limited number of studies have evaluated the evolutionary rate of the ORF1 gene. We found a time-scaled evolutionary tree of the complete ORF1 similar to that observed using GII.P16/GII.2 strains (2.03 × 10^–3^ s/s/y) (Nagasawa et al., 2018). Ozaki et al. (2018) estimated the evolutionary rate at 2.82 × 10^–3^ s/s/y for strains of the GII genogroup considering RdRp; however, each genotype has an evolutionary mechanism. Evolutionary analyses may vary depending on the genotype and the database used (Ozaki et al., 2018). The evolutionary rate found in our study is consistent for a conserved region of the genome such as ORF1. Viral genes involved in replication tend to undergo fewer changes in their proteins compared to the viral capsid. Thus, any change in these genes can have a major impact on viral fitness as proposed for the GII.P16/GII.4 strains.

While in this study we removing identical sequences to decrease the computational time-consuming, creating a selection-bias, we possibly ignored important temporal information. This limitation may have influenced to fully recapitulate the evolutionary history of GII.P16 sequences, including the tMRCA values. Nagasawa et al. (2018) found a common ancestor for the GII.P16/GII.2 strain detected between 2016 and 2017 in 1999, 17 years earlier that when they predominated. The only study that analyzed the tMRCA of GII.P16/GII.4 strains found values 2 years later (2013) (Ruis et al., 2017) of those obtained in this study (2011), which reinforces the importance of conducting more studies like this one. This difference may be linked to the non-random selection of sequences, however, more studies should be conducted to clarify the ancestry of this recombinant.

The GII.P16 genotype has already been associated with several different capsid genotypes (GII.P16-GII.2, GII.P16-GII.3, GII.P16-GII.4, GII.P16-GII.1, GII.P16-GII.12, GII.P16-GII.10) (Ruis et al., 2017; Nagasawa et al., 2018; Barclay et al., 2019). Previous studies suggested that the GII.4_Sydney strain acquired GII.P16 from the GII.P16-GII.2 strains (Tohma et al., 2017; Lun et al., 2018). This hypothesis was supported in this study, since strains clustered closer in the temporal reconstruction tree, and the TMRCA of both strains emerged in 2010. In conclusion, the results obtained strongly suggest that the recombinant GII.P16/GII.4 originated from the strains GII.P16/GII.2 and GII.P31/GII.4_Sydney.

In recent years, most studies on norovirus immunogenicity have focused on the antigenic sites involved in the blocking of HBGA carbohydrates, namely A-G (Debbink et al., 2012; Tohma et al., 2019). Several studies have shown that amino acid changes in these epitopes result in the ability to escape from the action of the immune system, leading to global epidemics, as demonstrated in the GII.4 Sydney_2012 lineage. In this study, we analyzed the epitopes (A-G), extensions of previously predicted and new uncharacterized antigenic sites proposed by Tohma et al. (2019). The GII.P16/GII.4 strains presented the same changes in amino acids 310, 333, 373, and 393 of the antigenic sites in the P2 subdomain already described previously among the GII.P16_GII.4 strains (Figure 3) (Cannon et al., 2017; Ruis et al., 2017), which were not presented in the reference GII.P31/GII.4_Sydney_2012 strain. Some of these changes seem to be very frequent in GII.P31/GII.4 strains. These corroborates with previous studies that observed the new GII.P16 polymerase recombinant with GII.4 capsid as a component of viral fitness, which might have influenced the transmissibility and spread of this virus (Ruis et al., 2017; Tohma et al., 2019).

Therefore, the cause of the emergence of this strain in several countries could be changes occurring in the ORF1 region. We found AA changes in non-structural proteins such as p48, p22, and RdRp that could directly affect viral replication. Considering specifically p22 and p48, diverse pathways can be altered, as these proteins have a role in blocking the host secretory pathway, which avoids cytokine secretion and the complement system cascade (Roth and Karst, 2016). Considering that noroviruses have high recombination rates and that the GII.4 genotype was prevalent in several places for a long time, additional and continuous evolutionary analyses of this genotype should be needed in the future.

While in this study we sequenced only samples from two states, making it difficult to fully recapitulate the evolutionary history of these pandemic viruses in Brazil, we present the first complete (or nearly complete) genome sequences of GII.P16/GII.4 viruses in Brazil. This information could facilitate the understanding of geographical distribution of this genotype at global level.

## Data Availability Statement

The datasets presented in this study can be found in online repositories. The names of the repository/repositories and accession number(s) can be found in the article/ Supplementary Material.

## Ethics Statement

The studies involving human participants were reviewed and approved by the Ethics Committee on Human Research of Evandro 117 Chagas Institute, Brazilian Ministry of Health (protocol No. 0039/2011 and protocol No. 118 0017/2014 update No. 1.318.103 of 2015). Written informed consent to participate in this study was provided by the participants’ legal guardian/next of kin.

## Author Contributions

JH contributed to all sections, including laboratory tests, molecular analyses, and writing the manuscript. LS revised the manuscript and performed the study design. TR wrote and revised the manuscript. ES and JC performed phylogenetic analysis of the sequences and all the bioinformatics approaches. AP and CL participated in laboratory analyses. ML and DT processed the clinical samples. YG and MN contributed to data analysis and the editing of the manuscript. All authors read and approved the final manuscript.

## Conflict of Interest

The authors declare that the research was conducted in the absence of any commercial or financial relationships that could be construed as a potential conflict of interest.

## Footnotes

1 http://www.bioinformatics.babraham.ac.uk/projects/trim_galore/
