# Supplementary material for: Evolutionary and Molecular Analysis of Complete Genome Sequences of Norovirus From Brazil: Emerging Recombinant Strain GII.P16/GII.4
Source: Front Microbiol. 2020 Aug 6;11:1870. doi: 10.3389/fmicb.2020.01870 (PMC7423841; doi:10.3389/fmicb.2020.01870)
Supplement: Supplementary file 1 [file Data_Sheet_1.docx]

**Table S1**. Sequencing information of the analyzed samples. Reads for Norovirus: refers to reads with match for norovirus in GenBank database. Coverage of bases (mean) for NoV: average coverage for each base in the norovirus sequence.

| Sample | Raw Data | Reads for Norovirus | Coverage of bases (mean) for NoV |
| --- | --- | --- | --- |
| JH 11 | 9,850,630 | 1,359 | 69 |
| JH12 | 7,356,558 | 2,579 | 76 |
| JH13 | 7,534,652 | 70,461 | 1,614 |
| JH15 | 12,350,976 | 155 | 1.9 |
| JH16 | 10,276,550 | 5,012 | 94.2 |
| JH17 | 11,059,584 | 142 | 3.4 |
| JH19 | 7,435,530 | 8,015 | 146,9 |
| JH21 | 9,253,080 | 507 | 13,9 |
| JH22 | 13,933,150 | 9,028 | 136 |
| JH23 | 13,212,682 | 138,002 | 2,688 |
| JH2 | 10,205,186 | 43,243 | 1,014 |
| JH30 | 11,119,928 | 47 | 1.3 |
| JH34 | 16,458,371 | 11,707 | 352 |
| JH37 | 15,339,492 | 68,776 | 6,386 |
| JH38 | 14,886,791 | 23,899 | 749 |
| JH40 | 15,319,487 | 1,989 | 61 |
| JH5 | 11,488,544 | 1,327 | 21 |
| JH6 | 10,611,122 | 11,628 | 227 |
| JH28 | 11,404,450 | 828 | 19 |
| JH29 | 11,104,876 | 5,258 | 123 |

Table S2. Amino acid changes in the ORF1 sites of GII.P16/GII.4 strains circulating in Brazil. The accession number of the prototype strain representative of the GII.P16/GII.4 genotype used as a reference was LC175468 (Hu/GII/JP/2016/GII.P16_GII.4_Sydney2012/Kawasaki194). In bold, amino acid changes have not yet been described in the literature.

| **Protein** | **AA change** | **Change of chemical nature and polarity** |
| --- | --- | --- |
| p48 | N9T | No |
| p48 | Y14C | No |
| p48 | S47P | Yes |
| **p48** | **D51G** | Yes |
| p48 | N52E | No |
| p48 | S53P | Yes |
| **p48** | **I83V** | No |
| p48 | K165R | No |
| p48 | N223S | No |
| p48 | E327G | Yes |
| **p48** | **L331F** | No |
| **NTPase** | **A399V** | No |
| NTPase | S644P | Yes |
| p22 | R731K | No |
| p22 | K750R | No |
| **p22** | **N751S** | No |
| p22 | I753T | Yes |
| **p22** | **G774S** | Yes |
| p22 | I786V | No |
| p22 | R809K | No |
| p22 | P845Q | Yes |
| p22 | A853T | Yes |
| Vpg | R887K | No |
| Vpg | V948I | No |
| Pro | V1057I | No |
| **Pro** | **I1080V** | No |
| **RDRP** | **A1091V** | No |
| **RDRP** | **V1175I** | No |
| **RDRP** | **E1323D** | No |
| RDRP | D1362E | No |
| RDRP | V1364I | No |
| RDRP | S1482T | No |
| RDRP | V1521I | No |
| RDRP | K1546Q | Yes |
| RDRP | T1549A | Yes |
| **RDRP** | **K1646R** | No |

**Table S3**. Path Sampling (PS) and stepping-stone sampling (SS) parameters used in the evolutionary analysis.

|  | | Strict | | | Relaxed | | |  |
| --- | --- | --- | --- | --- | --- | --- | --- | --- |
|  |  | **Constant**  **size** | **Bayesian**  **skyline** | **GMRF bayesian_**  **skyride** | **Constant size** | **Bayesian**  **skyline** | **GMRF**  **bayesian_skyride** | MLE |
| ps | ORF1 | -25467.5404196905 | -25457.0535759005 | -25523.4970602985 | -25393.3238110666 | -25382.8528369297 | -25425.3408017956 |  |
|  |  | **Constant**  **size** | **Bayesian**  **skyline** | **GMRF bayesian_**  **skyride** | **Constant size** | **Bayesian**  **skyline** | **GMRF**  **bayesian_skyride** |  |
| ss | ORF1 | -25467.8975 | -25457.29336 | -25524.76949 | -25393.57175 | -25384.07706 | -25424.24071 |  |
|  | | Strict | | | Relaxed | | |  |
|  |  | **Constant**  **size** | **Bayesian**  **skyline** | **GMRF bayesian_**  **skyride** | **Constant size** | **Bayesian**  **skyline** | **GMRF**  **bayesian_skyride** | ln BF |
| ps | ORF1 | 0 | -20.97368758 | **111.9132812** | -148.4332172 | -169.3751655 | -84.39923579 |  |
|  |  | **Constant**  **size** | **Bayesian**  **skyline** | **GMRF bayesian_**  **skyride** | **Constant size** | **Bayesian**  **skyline** | **GMRF**  **bayesian_skyride** |  |
| ss | ORF1 | 0 | -21.20827953 | **113.7439894** | -148.6514947 | -167.6408661 | -87.3135776 |  |
